# Validity of a dish composition database for estimating protein, sodium and potassium intakes against 24 h urinary excretion: comparison with a standard food composition database

**DOI:** 10.1017/S1368980019003550

**Published:** 2020-06

**Authors:** Nana Shinozaki, Kentaro Murakami, Shizuko Masayasu, Satoshi Sasaki

**Affiliations:** 1 Department of Social and Preventive Epidemiology, Graduate School of Medicine, The University of Tokyo, Tokyo, Japan; 2 Department of Social and Preventive Epidemiology, School of Public Health, The University of Tokyo, 7-3-1 Hongo, Bunkyo-ku, Tokyo 113-0033, Japan; 3 Ikurien-naka, Ibaraki, Japan

**Keywords:** Dish composition database, Food composition database, Validity, Dietary record, Urine collection

## Abstract

**Objective:**

We assessed the validity of a recently developed dish composition database (DCD) against urinary biomarkers compared with a standard food composition database (FCD).

**Design:**

Intakes of protein, Na and K were estimated by 2 × 24 h urine collections and by 4 d dietary record data based on the DCD (including 128 dishes) or FCD (including 1878 foods).

**Setting:**

Japan.

**Participants:**

A total of 161 men and 163 women aged 20–69 years.

**Results:**

Compared with the 24 h urine-based estimates, the median intakes estimated using the DCD and FCD differed significantly for protein and Na in men and for Na and K in women. Deattenuated Spearman correlation coefficients using 24 h urine-based estimates for the intakes of protein, Na and K were lower in the DCD (respectively: 0·26, 0·15 and 0·44 in men; 0·22, 0·27 and 0·22 in women) than those in the FCD (respectively: 0·43, 0·40 and 0·59 in men; 0·33, 0·45 and 0·42 in women). When data on dish portion size reported by participants were used for estimation instead of standard portion-size data based on the DCD, the accuracy of the estimated median intakes did not change notably, whereas the deattenuated correlation coefficients improved (for protein, Na and K respectively: 0·32, 0·31 and 0·56 in men; 0·31, 0·41 and 0·39 in women).

**Conclusions:**

The DCD supported by individual-level information on dish portion size showed fair to moderate validity in ranking individuals according to their intakes of protein, Na and K, similar to the FCD.

Obtaining accurate information on dietary intakes is essential for studies of diet and health. Most studies evaluate dietary intakes based on self-reported dietary assessment methods such as dietary records (DR) and 24 h dietary recalls (24-HDR)^(^^1^^)^. In DR and 24-HDR, participants are asked to record or recall the types and amounts of all foods and beverages consumed^(^^2^^)^. Although detailed information on food intake is necessary to calculate energy and nutrient intakes using a food composition database (FCD)^(^^3^^,^^4^^)^, recording or weighing all food items requires participant time and patience^(^^2^^,^^5^^–^^7^^)^. Moreover, since many foods are eaten as composite dishes prepared with other foods^(^^8^^,^^9^^)^, it is difficult to describe the details of all single ingredients in ready-to-eat or restaurant meals. Such food-based dietary assessment methods are especially challenging for those lacking knowledge of foods or cooking^(^^9^^)^.

To overcome these issues, dish-based dietary assessment instruments such as dish-based DR^(^^6^^,^^10^^–^^12^^)^ and dish-based FFQ^(^^9^^,^^13^^–^^19^^)^ have been developed in several countries. These methods require information on the intakes of mixed dishes instead of detailed information on the intakes of individual food ingredients in cooked dishes to estimate nutrient intakes from diet. The nutrient intakes from dishes are usually calculated using dish composition databases (DCD). Such dish-based methods have potential advantages over conventional food-based methods in dietary surveys because they may reduce the burden on survey participants and staff by omitting the processes of providing detailed food information or disaggregating information on mixed dishes into individual food ingredients^(^^10^^–^^12^^)^. Moreover, a DCD is also useful in conventional food-based dietary surveys by standardizing the methods used to estimate dietary intake from mixed dishes, as well as by complementing information on ingredients in dishes not obtained from participants^(^^20^^–^^22^^)^. Furthermore, since mixed dishes represent a combination of foods and cooking methods, assessment of diet based on dishes and recipes rather than on individual food ingredients may be instrumental for characterizing the dietary patterns of populations^(^^23^^)^.

Given that Asian cuisine consists primarily of a variety of mixed dishes cooked with many ingredients, seasonings and oils^(^^5^^,^^6^^,^^10^^,^^11^^,^^14^^,^^17^^–^^19^^,^^24^^)^, food-based approaches may increase the burden and reporting error in these populations^(^^5^^)^. Hence, a dish-based approach is recommended as a new strategy to improve dietary assessment, especially in countries with Asian diets^(^^5^^)^. Several studies have developed dish-based dietary assessment methods or DCD in Asian countries, including Japan^(^^6^^,^^9^^–^^12^^,^^25^^–^^27^^)^, South Korea^(^^15^^,^^24^^)^ and Bangladesh^(^^18^^)^. These studies assessed the validity of the dish-based dietary assessment tools using a food-based DR as a reference. Among these tools is a recently developed DCD for Japanese people based on a 4 d DR obtained from 252 men and women^(^^25^^)^. This DCD was developed for application to dietary surveys in Japan because the Japanese standard FCD has a limited number of dish items^(^^28^^)^. The ability of this DCD to estimate food group and nutrient intakes was compared with that of the Japanese standard FCD^(^^28^^)^ using a 4 d DR. The Spearman correlation coefficients between the two databases were 0·65, 0·46 and 0·69 for intakes of protein, Na and K, respectively, in men. The respective values in women were 0·53, 0·46 and 0·55. However, this is not an appropriate method for validation because the reference is self-reported dietary information, which may be biased and have correlated errors with dietary intakes assessed based on dishes^(^^29^^–^^31^^)^. A stricter validation assessment requires an objective measure of dietary intake as the reference^(^^5^^,^^29^^–^^34^^)^. However, to our knowledge, the validity of dietary intake estimated based on DCD has not yet been evaluated using biomarkers.

The aim of the present study was to assess the validity of the intakes of protein, Na and K estimated using the DCD^(^^25^^)^ against 24 h urine excretion in a Japanese population. The validity was also compared with a food-based method using the Japanese standard FCD^(^^28^^)^.

## Methods

### Data source and basic characteristics

The current cross-sectional study was based on data from a nationwide dietary survey conducted in twenty study areas consisting of twenty-three prefectures in Japan between February and March 2013. Details of the survey have been published elsewhere^(^^35^^,^^36^^)^. Briefly, 199 dietitians working at separate welfare facilities were recruited as research dietitians and then they recruited their co-workers as well as family members of the co-workers as survey participants. Each study area included approximately four apparently healthy individuals (two men and two women) from each of five 10-year age groups (20–29, 30–39, 40–49, 50–59 and 60–69 years). In total, 196 men and 196 women aged 20–69 years completed both a four-non-consecutive-day weighed DR and two 24 h urine collections (UC). Information on age, educational background and smoking status was obtained via questionnaire responses. Body weight (nearest 0·1 kg) and height (nearest 0·1 cm) were measured in light clothes and without shoes following standardized procedures. BMI was calculated as body weight (in kilograms) divided by the square of body height (in metres). The measurement schedule was arranged such that all measurements were completed within 10–14 d, with a mean of 9 d.

### Dietary assessment

DR were conducted for four non-consecutive days, considering within-person variation in dietary intakes and feasibility of recording diet. Details of the DR have been published elsewhere^(^^36^^)^. Briefly, the participants were asked to record all foods and beverages consumed for three working days and one non-working day, excluding days before and after a night shift. Research dietitians explained to the participants how to keep the DR and asked them to weigh foods and beverages with a provided digital scale or measuring spoon and cup. The main items recorded on the DR sheets were dish names, food names (including beverages and any ingredients in dishes) and the approximate amounts (amount measured by measuring spoon or cup, or the number of foods (e.g. two slices of bread)) or measured weights of foods and dishes consumed. The record sheets were submitted to a research dietitian at each facility shortly after recording and then checked by the research dietitian as soon as possible. If there was missing or ambiguous information on the name or amount of foods on the sheet, the research dietitian asked the participant directly.

The research dietitian at each facility coded the records using the FCD^(^^28^^)^ in a uniform procedure. Each item recorded in the column containing the names of the foods (91 045 items) was assigned an FCD food code. The FCD includes 1878 food codes, 99·1 % of which (1862 items) consist of a single food item. Therefore, almost all recorded items were coded by a single food code. All food codes and weights were then reconfirmed by two other research dietitians at the central office of the study.

A registered dietitian not involved in the coding of the FCD coded the records using the DCD^(^^25^^)^. A detailed description of the development process and structure of the DCD has been given elsewhere^(^^25^^)^. Briefly, the DCD was developed based on non-consecutive 16 d weighed DR completed by healthy Japanese men (*n* 126) and women (*n* 126). This DR included 71 213 dishes in the ‘dish name’ column. They were aggregated into 128 types of dishes based on the similarities in dish name, ingredients, cooking methods, and energy and nutrient contents. Each of the 128 dish types was then named and assigned a dish code. The mean of the weight of dishes categorized into the same dish code was calculated as the standard portion size (PS) of that dish. Similarly, the mean food group and nutrient contents were calculated for each dish code.

A total of 26 642 dishes were included in the ‘dish name’ column in the DR in the present study. All dishes were assigned to the 128 dish codes in the DCD based on their names. Supplementary information on dishes, if any (e.g. brand or manufacturer names), recorded in the DR was also used to code the dishes. If there was no corresponding dish name in the DCD, we assigned it the code of a dish that was similar in ingredients, dish type or cooking method to a dish included in the dish names.

### 24 h Urine collections

Two 24 h UC were collected on non-consecutive days. To reduce participant burden and to ensure quality of the DR and UC, UC was planned one before and another after the four DR days^(^^37^^)^. Briefly, the participants were asked to collect all urine voided during a 24 h period and to record the start and finish times of the collection periods. Research dietitians explained the procedure of UC to the participants and asked participants not to keep the collected urine in extremely high or low temperatures. They were also asked to record the estimated volume of missing urine specimens if they failed to collect urine. The 24 h urine volume was adjusted by the self-reported collection time and missing urine volume. This adjustment method was validated using the *p*-aminobenzoic acid check method^(^^38^^)^. Urine samples were carefully mixed and aliquoted into a 10 ml container by the research dietitian. The urine sample was stored in a cold reserving box with refrigerants, or in a freezer when it was difficult to collect the urine sample from each facility soon. The collected urine was transferred to and analysed by LSI Medience Corporation (Tokyo, Japan). Urinary creatinine concentrations were determined by enzyme methods using the Iatro-LQ CRE (A) II assay (LSI Medience Corporation). Urinary urea-N was determined by the urease and leucine dehydrogenase method using the Iatro-LQ UN rate (A) II assay (LSI Medience Corporation). Urinary Na and K concentrations were determined by the electrode method using a JCA-BM6050 clinical chemistry analyser (JEOL Ltd, Tokyo, Japan). The total 24 h excretion was calculated by multiplying the measured concentration by the volume of 24 h urine. Urinary urea-N (g/d) was divided by 0·85 (proportion of urinary urea-N to total urinary N excretion) and further divided by 0·81 (urinary excretion rate) to convert the measurement to dietary N and then multiplied by 6·25 to obtain the dietary protein intake (g/d)^(^^39^^,^^40^^)^. Urinary Na and K (mg/d) were converted to the corresponding dietary intakes (mg/d) by dividing by urinary excretion rates (0·86 and 0·77, respectively)^(^^41^^)^.

### Data analysis

A total of 392 participants completed both the 4 d DR and two UC. The completeness of the UC was assessed by calculating the ratio of observed to expected creatinine excretions as follows: 100 × [urinary creatinine (mmol/d) × 113]/[*C* × body weight (kg)], where *C* = 24 for men and 21 for women^(^^35^^,^^38^^,^^42^^)^. A total of sixty-six participants with ratio of <60 % or >140 % were excluded. We further excluded two participants with UC with insufficient measurement intervals (<3 d)^(^^35^^)^. Finally, 161 men and 163 women were included in the analysis.

The statistical analyses were conducted for men and women separately. The mean intakes of protein, Na and K were estimated by the 4 d DR using the FCD and DCD. In the calculation of dietary intake using the DCD, the amount of a dish consumed was replaced with the standard PS for that dish in the DCD. In addition, to evaluate the effect of the use of the PS of dishes reported by the participants instead of the standard PS on the validity of the DCD, we also computed the nutrient intakes from the DCD adjusted for the reported PS of the dish in the DR. The values were calculated as nutrient content in the dish in the DCD (g) multiplied by the ratio of the reported PS of the dish in the DR (g) to the standard PS of the dish in the DCD (g).

Nutrient intakes estimated using the FCD and the DCD were compared with those estimated from the mean of two 24 h urinary excretions as reference. First, the median nutrient intakes derived from the FCD and DCD were compared with those estimated by the UC using Wilcoxon signed-rank tests. Next, the ranking abilities of the FCD and DCD were evaluated using Spearman correlation coefficients with the UC-based estimates. In order to correct for within-person variation in the UC that would reduce the correlation coefficient towards zero, we also calculated deattenuated correlation coefficients using the following formula^(^^43^^)^: 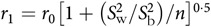, where *r*_1_ is the deattenuated correlation coefficient, *r*_0_ is the crude correlation coefficient, 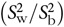 is the ratio of the within- to between-person variance and *n* is the number of UC. Additionally, a Bland–Altman plot was used to assess the agreement of the values estimated using the FCD and DCD and those estimated using the UC^(^^44^^,^^45^^)^. A regression analysis was used to detect the proportional error between the two methods. All analyses were conducted using the statistical software package SAS version 9.4. Two-sided *P* values < 0·05 were considered statistically significant.

## Results

Table 1 shows the basic characteristics of the participants. The mean age was 45·2 years in men and 44·4 years in women; the mean BMI was 23·8 and 22·3 kg/m^2^ in men and women, respectively. The self-reported energy intake estimated from each food using the FCD was 9971 kJ/d for men and 7998 kJ/d for women.

**Table 1:** Basic characteristics of the participants: Japanese men and women aged 20–69 years (*n* 324)

Variable	Men (*n* 161)		Women (*n* 163)	
	Mean or *n*	sd or %	Mean or *n*	sd or %
Age (years), mean and sd	45·2	13·1	44·4	13·1
Body height (cm), mean and sd	170·3	5·5	157·4	5·7
Body weight (kg), mean and sd	69·2	10·7	55·3	8·6
BMI (kg/m^2^), mean and sd	23·8	3·2	22·3	3·3
Educational background, *n* and %				
Junior or senior high school	35	21·7	59	36·2
Vocational school or junior college	44	27·3	69	42·3
University or graduated school	82	50·9	34	20·9
Others	0	0·0	1	0·6
Smoking status, *n* and %				
Never	59	36·7	129	79·1
Past	49	30·4	12	7·4
Current	53	32·9	22	13·5
Reported dietary intake*, mean and sd				
Energy (kJ/d)	9971	1916	7998	1490
Protein (% of energy)	13·9	2·1	14·4	2·1
Fat (% of energy)	26·8	4·8	29·9	5·1
Carbohydrate (% of energy)	52·9	7·7	53·2	5·3

* Calculated from each food in the 4 d dietary record using the Standard Tables of Food Composition in Japan^(^^28^^)^.

Table 2 shows the median intakes of protein, Na and K estimated by the UC, FCD, DCD and DCD with reported PS. For protein, significant differences were observed between UC and the FCD and between UC and the DCD in men, and between UC and the DCD with reported PS in women. For Na, all values estimated using the FCD, DCD and DCD with reported PS differed significantly from the UC estimates in both sexes. For K, significant differences were observed between UC and the DCD with reported PS in men, and between UC and respective values estimated using the FCD, DCD and DCD with reported PS in women.

**Table 2:** Intakes of protein, sodium and potassium estimated by two 24 h urine collections (UC), the food composition database (FCD), the dish composition database (DCD) and the dish composition database with reported portion size (DCD with reported PS) in Japanese men (*n* 161) and women (*n* 163) aged 20–69 years

	Protein (g/d)				Na (mg/d)				K (mg/d)			
	Median	P_25_	P_75_	*P* *	Median	P_25_	P_75_	*P* *	Median	P_25_	P_75_	*P* *
Men												
Two 24 h UC†	89·8	79·5	103·5	–	5240	4360	6595	–	2602	2096	3095	–
FCD‡	82·9	69·7	93·2	<0·0001	4442	3588	4895	<0·0001	2667	2263	3231	0·14
DCD§	74·7	64·0	85·5	<0·0001	5017	4151	5903	0·03	2550	2157	3011	0·52
DCD with reported PS‖	88·2	73·6	99·8	0·07	5635	4626	6846	0·03	2881	2464	3391	<0·0001
Women												
Two 24-h UC†	68·3	59·4	79·5	–	4442	3520	5311	–	2305	1794	2768	–
FCD‡	66·9	58·6	76·3	0·41	3586	2932	4265	<0·0001	2560	2169	2878	0·0002
DCD§	72·4	60·5	83·0	0·06	4765	3857	5848	0·01	2595	2175	3037	0·0002
DCD with reported PS‖	72·3	62·8	82·5	0·009	5018	3861	5930	0·0006	2652	2254	3017	<0·0001

P_25_, 25th percentile; P_75_, 75th percentile.

* Differences from values derived from the two 24 h UC were tested by Wilcoxon signed-rank tests.

† Nutrient intake based on the mean of two 24 h UC was calculated as follows: protein intake (g/d) = 24 h urinary urea-N (g/d)/(0·85 × 0·81) × 6·25^(^^39^^,^^40^^)^; Na intake (mg/d) = 24 h urinary Na (mg/d)/0·86^(^^41^^)^; and K intake (mg/d) = 24 h urinary K (mg/d)/0·77^(^^41^^)^.

‡ Estimated from each food in the 4 d dietary record using the Standard Tables of Food Composition in Japan^(^^28^^)^.

§ Estimated from each dish in the 4 d dietary record using the DCD with standard PS (weight of each dish in the DCD)^(^^25^^)^.

‖ Calculated by adjusting the nutrient content of a dish in the DCD by the reported PS of the dish in the 4 d dietary record. The calculation method was as follows: estimated nutrient intake from a dish adjusted by the reported PS = nutrient content of the dish in the DCD (g) × reported PS of the dish in the dietary records (g)/standard PS of the dish in the DCD (g).

Table 3 shows Spearman correlation coefficients between UC and the FCD, DCD and DCD with reported PS for estimates of protein, Na and K intake. For both sexes, the crude correlation coefficients for protein, Na and K were highest in the FCD, followed by the DCD with reported PS, then the DCD. The deattenuated correlation coefficients were 1·1–1·3 times higher than the crude values for the respective nutrients.

**Table 3:** Spearman correlation coefficients between two 24 h urine collections* and the food composition database (FCD), the dish composition database (DCD) and the dish composition database with reported portion size (DCD with reported PS) for estimates of protein, sodium and potassium intakes in Japanese men (*n* 161) and women (*n* 163) aged 20–69 years

	Protein intake				Na intake				K intake			
	Crude		Deattenuated		Crude		Deattenuated		Crude		Deattenuated	
	*r*	*P*	*r*	*P*	*r*	*P*	*r*	*P*	*r*	*P*	*r*	*P*
Men												
FCD†	0·37	<0·0001	0·43	<0·0001	0·32	<0·0001	0·40	<0·0001	0·48	<0·0001	0·59	<0·0001
DCD‡	0·22	0·004	0·26	0·0008	0·12	0·13	0·15	0·06	0·36	<0·0001	0·44	<0·0001
DCD with reported PS§	0·27	0·0005	0·32	<0·0001	0·25	0·002	0·31	0·0001	0·45	<0·0001	0·56	<0·0001
Women												
FCD†	0·28	0·0002	0·33	<0·0001	0·36	<0·0001	0·45	<0·0001	0·37	<0·0001	0·42	<0·0001
DCD‡	0·19	0·02	0·22	0·005	0·22	0·004	0·27	0·0004	0·20	0·01	0·22	0·004
DCD with reported PS§	0·26	0·0008	0·31	0·0001	0·33	<0·0001	0·41	<0·0001	0·34	<0·0001	0·39	<0·0001

* Nutrient intake based on the mean of two 24 h urine collections was calculated as follows: protein intake (g/d) = 24 h urinary urea-N (g/d)/(0·85 × 0·81) × 6·25^(^^39^^,^^40^^)^; Na intake (mg/d) = 24 h urinary Na (mg/d)/0·86^(^^41^^)^; and K intake (mg/d) = 24-h urinary K (mg/d)/0·77^(^^41^^)^.

† Estimated from each food in the 4 d dietary record using the Standard Tables of Food Composition in Japan^(^^28^^)^.

‡ Estimated from each dish in the 4 d dietary record using the DCD with standard PS (weight of each dish in the DCD)^(^^25^^)^.

§ Calculated by adjusting the nutrient content of a dish in the DCD by the reported PS of the dish in the 4 d dietary record. The calculation method was as follows: estimated nutrient intake from a dish adjusted by the reported PS = nutrient content of the dish in the DCD (g) × reported PS of the dish in the dietary records (g)/standard PS of the dish in the DCD (g).

**Fig. 1:**
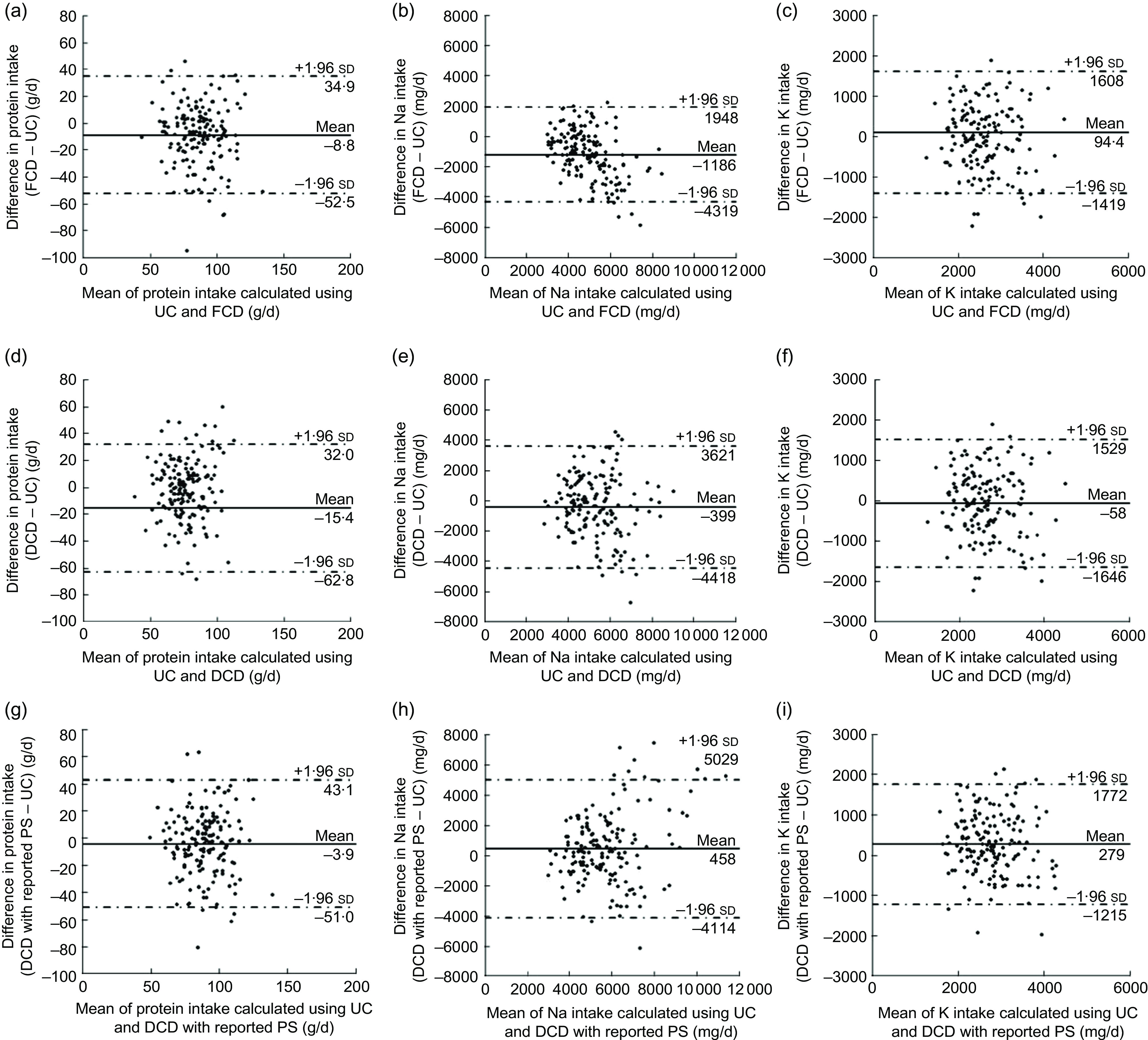
Bland–Altman plots assessing the agreement of the estimated intakes of protein, sodium and potassium between 24 h urine collections (UC) and (a–c) the food composition database (FCD), (d–f) the dish composition database (DCD) and (g–i) the dish composition database with reported portion size (DCD with reported PS) in Japanese men aged 20–69 years (*n* 161). —— represents the mean difference and – · – · – · – represent lower and upper 95 % limits of agreement

**Fig. 2:**
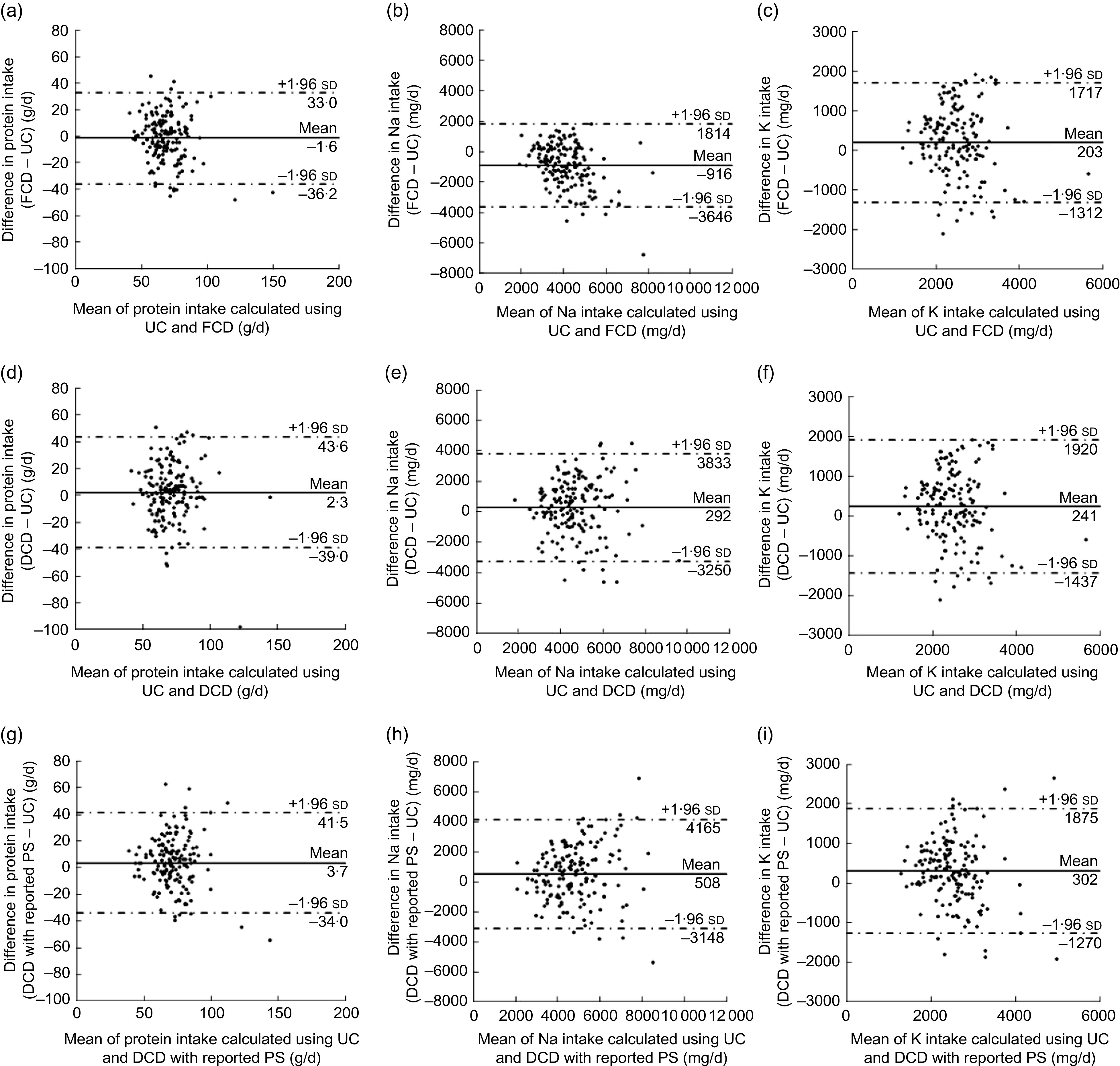
Bland–Altman plots assessing the agreement of the estimated intakes of protein, sodium and potassium between 24 h urine collections (UC) and (a–c) the food composition database (FCD), (d–f) the dish composition database (DCD) and (g–i) the dish composition database with reported portion size (DCD with reported PS) in Japanese women aged 20–69 years (*n* 163). —— represents the mean difference and – · – · – · – represent lower and upper 95 % limits of agreement

Bland–Altman plots were used to assess the agreement between UC and the FCD, DCD and DCD with reported PS for nutrient intakes (Fig. 1 for men and Fig. 2 for women). The limits of agreement were wide for all nutrients and methods, indicating poor agreement at the individual level. The absolute values of the mean differences were larger in the DCD with reported PS than those in the DCD, except for protein in men. The mean difference in Na intake was larger in the FCD than those in the DCD and DCD with reported PS. In men, regression analysis showed significant linear trends in Na intake estimated by the FCD (Fig. 1(b); slope = −0·659) and the DCD with reported PS (Fig. 1(h); slope = 0·410). In women, a significant linear trend was observed for protein and Na estimated by the FCD (Fig. 2(a); slope = −0·269 and Fig. 2(b); slope = −0·547, respectively). These trends indicated that the agreement in the estimations of intake between the methods varied according to the intake level.

## Discussion

In the present study, we assessed the validity of the DCD against two 24 h UC in comparison with the standard FCD. Our results showed that the DCD, DCD with reported PS and FCD had difficulty in accurately estimating the absolute intakes of protein, Na and K at individual and population levels. The deattenuated correlation coefficients with UC were fair to moderate, irrespective of the database used. Although the correlation coefficients with UC were lower in the DCD with standard PS than those in the FCD, the use of information on reported PS increased the correlation coefficients to the same level as those of the FCD, especially in women. To our knowledge, the present study is the first to assess the validity of the DCD for estimating nutrient intakes against 24 h urine excretion.

Compared with UC-based estimates, median intakes estimated using the DCD and FCD differed significantly for protein and Na in men and for Na and K in women. The DCD-based estimates adjusted by the reported PS also differed from the UC-based estimates except for protein in men. Moreover, the Bland–Altman plots showed poor agreement at the individual level for all nutrients and methods. Theoretically, the application of the DCD to the DR may result in measurement error by diluting detailed information on individual foods consumed by participants^(^^25^^)^. However, our results showed similarly low abilities of the FCD and DCD to estimate absolute nutrient intakes, indicating the difficulty in assessing intakes based on self-reported dietary data.

Self-reported dietary information is subject to measurement error caused by various factors, including within-individual variation in dietary intake^(^^1^^,^^33^^,^^46^^)^. In particular, DR require high motivation and literacy for participants and may also alter dietary behaviour^(^^7^^,^^47^^,^^48^^)^, and misreporting of protein, Na and K in Japanese has been reported^(^^48^^–^^50^^)^. Our results showed that Na intake was underestimated by the FCD and was less underestimated by the DCD. Moreover, the Bland–Altman plots showed that the FCD underestimated Na intake with increasing intakes. The intake of seasonings, which are the main contributors to Na intake^(^^36^^,^^51^^)^, may not always be correctly reported by participants in DR. This would cause measurement error in the FCD-based estimates, as they are affected by under- or over-reporting of all single food items such as cooking salt and seasonings. Meanwhile, the DCD-based estimates may be less likely to be affected by misreporting of certain food ingredients in mixed dishes in DR, since self-reported information on the types or amounts of each food is not used to estimate dietary intakes. Instead, the DCD would misestimate dietary intakes when the name or amount of dishes consumed is not correctly recorded in the DR.

The accuracy of the nutrient databases is another factor that may contribute to measurement error^(^^3^^)^. Although the nutrient content of foods in the standard FCD is derived from chemical analysis^(^^28^^)^, the values vary by inherent, environmental, processing and analytical factors^(^^3^^)^. The same is true for the DCD, since the nutrient contents of each dish in the DCD were calculated from its individual ingredients using the FCD^(^^25^^)^. In addition, the DCD may have other sources of measurement error. For instance, the DCD was developed based on dietary information from DR conducted in different areas, seasons and years, and among a population with a different mean age^(^^25^^)^. This may lead to differences in PS or dish composition between the data in the DCD and dishes consumed by the study participants. Furthermore, men generally eat larger amounts of food than women, whereas the DCD was developed for men and women combined. Our results showed that the DCD-based estimates for three nutrients tended to be underestimated in men and overestimated in women. This systematic error should be taken into consideration when interpreting nutrient intakes estimated using the DCD.

Theoretically, the measurement error in estimating nutrient intakes caused by differences in PS between the DCD and actual diets can be eliminated by using information on reported PS. However, the Bland–Altman plots showed that the use of reported PS increased the differences in absolute intakes between the DCD and UC at the individual level for Na and K. Therefore, a major factor causing measurement error in the DCD is the difference in dish composition between the DCD and diets consumed. Additionally, when reported PS was used, the absolute Na intake was overestimated with increasing intake in men. This finding suggests that the actual Na intake did not increase as the PS of dishes increased. Considering that overestimation of Na by the DCD with reported PS was also observed in median intake estimation in men and women, adjustment using reported PS may not be beneficial in the estimation of absolute intake of Na.

The correlation coefficients with UC were fair to moderate in the DCD and DCD supported by reported PS, as well as the FCD. The fair correlation between UC and DR was also observed for Na (0·12) and K (0·33) in a previous study^(^^52^^)^. Taken together with the low validity of both the DCD and FCD in median intake estimation, it may be difficult to accurately assess absolute intakes of Na, K and protein using DR, whichever database is used.

The DCD showed lower correlation coefficients with UC than did the FCD. This may be because the between-person variations in nutrient intakes were uniformly diluted by allocating standard PS in the DCD to all participants. However, the DCD with reported PS had relatively comparable correlation coefficients to those of the FCD, indicating that the DCD can perform similarly to the FCD in ranking individuals according to their intakes of protein, Na and K if supported by information on the total weight of each dish consumed. While the DCD with reported PS showed a similar ranking ability to that of the FCD especially in women, the correlation coefficients between the DCD with reported PS and UC were similar between sexes. Therefore, there was no significant difference in the ranking ability of the DCD among men and women. In studies of diet–disease relationships, ranking participants according to their dietary intake is more important than estimating their absolute intake level^(^^43^^)^. To rank individuals based on dietary intake, food-based dietary assessment methods require detailed information on all single food items. Meanwhile, the use of the DCD enables the ranking of individuals if there is no information on the single ingredients in dishes. Accordingly, the DCD may contribute to the development of easier dietary assessment tools based on information on dishes (e.g. dish-based dietary questionnaires). However, information on the mass of dishes consumed in addition to dish name is necessary to rank individuals. Moreover, the method to name dishes or to select dish codes is subjective and differs by individual. In the present study, a registered dietitian assigned the dish codes. This may have led to an overestimation of the ability of the DCD because dietitians were likely to have more knowledge about food and cooking compared with the study participants. Conversely, the ability of the DCD may have been underestimated because the registered dietitian involved with coding did not eat or see that dish. In any case, validation studies using the dish codes selected by study participants are needed to determine the practical application of dish-based dietary assessment methods based on the DCD.

The current study has several limitations. First, although urinary biomarkers are often used as a reference for the validation of other dietary methods, they are affected by non-dietary sources of variations, including biological and analytical factors^(^^33^^,^^53^^)^. The difference in analytical methods between N in foods and urinary N may have added a bias in estimates of protein intake. In addition, although widely used, the coefficients used to calculate intakes of protein, Na and K from urinary biomarkers have not been validated in a Japanese population. The accuracy of these coefficients may affect the validity of the DCD and FCD in estimating absolute intake, not but in ranking individuals. Moreover, we could not assess the validity of energy intake using a biomarker, such as doubly labelled water, as it is an economically expensive and burdensome protocol^(^^2^^)^. Second, the difference in estimation ability between the DCD and FCD may be underestimated because the dietary intakes estimated were both based on the same DR. In the present study, we simulated a dish-based dietary assessment method by assigning dish codes of the DCD to the food-based DR. However, since participants’ burden of recording items may differ between dish-based DR and food-based DR, the degree of misreporting possibly differs between them in a real setting. Although a comparison between the DCD and FCD was not the main objective of the present study, the results should be carefully interpreted. Third, duplicate coding was not conducted when coding the DR. Although the food coding was rechecked by other dietitians, the dish coding was not checked by other dietitians due to limited manpower. This may have caused coding errors and eventually misestimation of the validity of the FCD and DCD. Duplicate coding may be necessary to reduce miscoding and evaluate the ability of the databases more rigorously. Finally, the participants may not represent the general Japanese population. They were not randomly selected and were volunteers who were likely to be more health conscious than the general population. Nevertheless, the weight and height of the participants were similar to those of the general population in Japan^(^^54^^)^.

## Conclusion

In conclusion, we assessed the validity of a recently developed DCD against urinary biomarkers compared with the standard FCD. For the absolute intakes of protein, Na and K, the validity of the DCD was low for median intake estimation and fair to moderate for ranking individuals by their intakes, similar to the FCD irrespective the use of information on reported PS of dishes. Although the ranking ability was lower in the DCD with standard PS than in the FCD, the use of information on reported PS increased the ranking ability of the DCD to the same level as the FCD, especially in women. Hence, the DCD may be a promising tool for dietary assessment, if supported by information about the PS of dishes consumed. The findings of the present study warrant further investigation of the practical utilization of the DCD in dietary surveys.
